# Skin Infections Caused by Panton-Valentine Leukocidin and Methicillin-Susceptible *Staphylococcus aureus* in Child, Japan

**DOI:** 10.3201/eid3106.241955

**Published:** 2025-06

**Authors:** Kensuke Shoji, Kazue Yoshida, Marie Takenouchi, Junzo Hisatsune, Shoko Kutsuno, Chika Arai, Kanako Masuda, Motoyuki Sugai, Takashi Ishikawa, Toshinao Kawai, Kazuhiro Uda, Isao Miyairi

**Affiliations:** National Center for Child Health and Development, Tokyo, Japan (K. Shoji, K. Yoshida, M. Takenouchi, T. Ishikawa, T. Kawai, K. Uda, I. Miyairi); National Institute of Infectious Diseases, Japan Institute for Health Security, Tokyo (J. Hisatsune, S. Kutsuno, C. Arai, M. Sugai); Project Research Center for Nosocomial Infectious Diseases, Hiroshima University, Hiroshima, Japan (J. Hisatsune, S. Kutsuno, C. Arai, K. Masuda, M. Sugai); Okayama University Hospital, Okayama, Japan (K. Uda); Hamamatsu University School of Medicine, Hamamatsu, Japan (I. Miyairi)

**Keywords:** Staphylococcus, Panton-Valentine leucocidin, methicillin-susceptible Staphylococcus aureus, MSSA, skin infections, pediatrics, Japan

## Abstract

We describe a pediatric case of recurrent skin infections caused by a Panton-Valentine leukocidin and exfoliative toxin E double-positive methicillin-susceptible *Staphylococcus aureus* clonal complex 188 clone. Most of the patient’s family members were infected with the same strain, and intrafamilial transmission was strongly suspected. Decolonization procedures were not effective.

Recurrent skin and soft tissue infections (SSTIs) caused by Panton-Valentine leukocidin (PVL)–positive methicillin-resistant *Staphylococcus aureus* (MRSA) have been reported worldwide (*1*,*2*). Recurrence may be attributed to intrafamilial transmission; in such cases, decolonization by nasal mupirocin ointment and topical skin application of 4% chlorhexidine are treatments for family members (*3*). Methicillin-susceptible *S. aureus* (MSSA) strains that possess virulence factors similar to those of MRSA have been reported (*4*,*5*); however, the clinical significance of such bacterial strains and the efficacy of decolonization are still unclear. We report a case of a child in Japan with recurrent SSTIs caused by PVL and exfoliative toxin E (ETE) double-positive MSSA refractory to decolonization, with evidence of intrafamilial transmission.

## The Study

**Figure 1:**
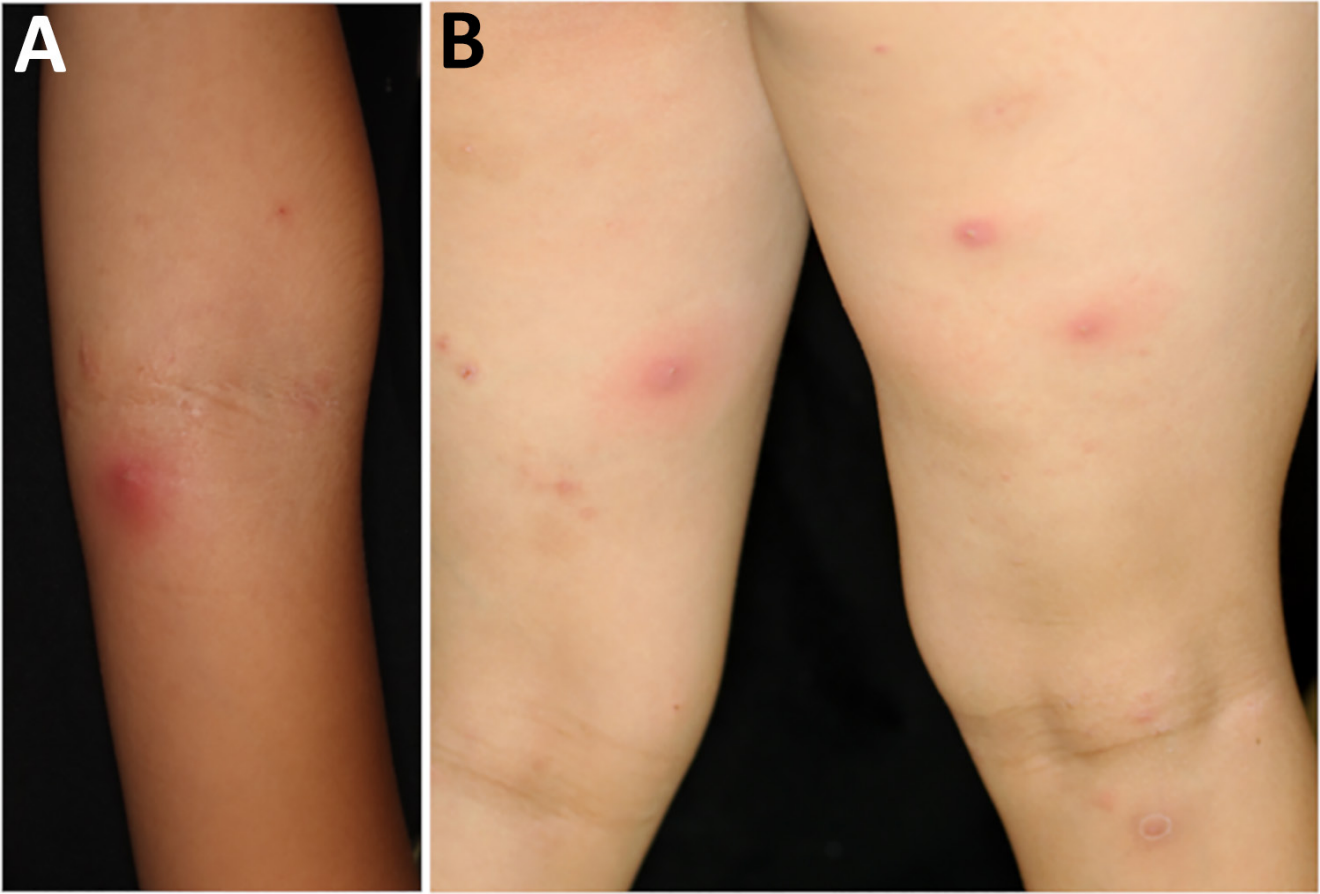
Multiple furuncles (furunculosis) on forearm (A) and thigh (B) of child experiencing recurrent skin infections caused by Panton-Valentine leukocidin and exfoliative toxin E double-positive methicillin-susceptible *Staphylococcus aureus* clonal complex 188 clone, Japan.

A 7-year-old girl with atopic dermatitis was referred to the National Center for Child Health and Development (Tokyo, Japan) for impetigo and recurrent multiple furuncles (furunculosis) (Figure 1). The child had no history of serious infections and no suspected immunodeficiencies. We instructed the patient to maintain skin cleanliness by showering and using moisturizers and to repeatedly treat with oral antimicrobial agents when furuncles manifested. However, the skin lesions relapsed 2–4 weeks after discontinuing the antimicrobial therapy. Culture of the pus from a furuncle identified MSSA (strain name JH62PP1) (Appendix Table). 

**Figure 2:**
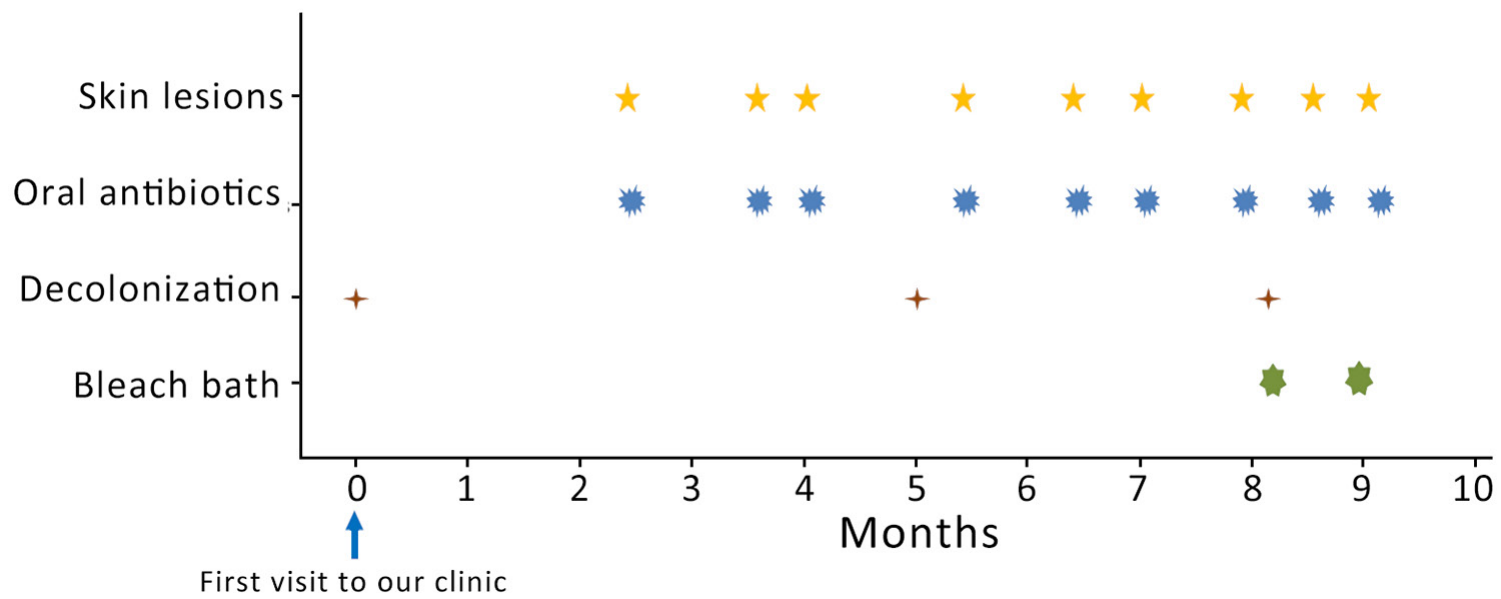
Timeline of symptoms and treatment of child experiencing recurrent skin infections caused by Panton-Valentine leukocidin and exfoliative toxin E double-positive methicillin-susceptible *Staphylococcus aureus* clonal complex 188 clone, Japan.

The patient’s father, older brother (12 years of age), and younger brother (3 years of age) also had similar skin lesions; we suspected intrafamilial infection of MSSA. In addition to lifestyle guidance (hand hygiene; keeping fingernails short; changing underwear, towels, and sleepwear each day; washing sheets and pillowcases weekly; washing the body with soap daily; and avoiding sharing personal items), all family members underwent decolonization using mupirocin ointment (applied to each nostril 3×/d for 3 days) and 4% chlorhexidine (applied to all skin areas below the neck 3×/wk for 4 weeks). Although the time interval between relapse of skin lesions increased temporarily, the frequency returned to the same level ≈4 months after the first decolonization (Figure 2). 

We then attempted decolonization for the patient and each family member with a bleach bath in water containing 0.005% hypochlorous acid as approved by the institutional ethics committee (NCCHD-1912). We instructed the patient and family to first wash their bodies with soap, then soak in the bathtub for 5–10 minutes, and finally rinse thoroughly in the shower. They performed the procedure twice a week. However, recurrences occurred within ≈2 weeks, indicating that the bath was not effective. 

We assessed the patient’s immune condition by blood tests, including a complete blood count with differential, immunoglobulin and complement levels, lymphocyte proliferation assay, superoxide production, and flow cytometry. The results of those tests were within reference ranges; we considered known primary immunodeficiencies, including chronic granulomatous disease and hyper-IgE syndrome, unlikely. Thereafter, we abandoned attempts at decolonization and gave the patient oral antimicrobial medication when a skin lesion formed. The frequency of skin lesions improved when the patient was 10 years of age; we observed no relapses after the patient turned 11, with no additional interventions. Outpatient follow-up ended.

In this case, we strongly suspected familial transmission of *S. aureus*. Therefore, we conducted a phylogenetic analysis using whole-genome sequencing on a total of 7 MSSA strains: 2 strains (JH62PP1 and JH62PP2) isolated from swab specimens of the patient’s pus at the initial and follow-up visits and 5 strains (JH62PN, JH62F, JH62M, JH62B1, and JH62B2) isolated from nasal swab specimens of the patient and 4 other family members before decolonization treatment. We performed purification of genomic DNA, preparation of sequencing libraries, whole-genome sequencing, and core-genome–based phylogenetic analyses in this study as previously described (*6*). We deposited raw read sequences obtained in this study in GenBank/DDBJ/EMBL (BioProject no. PRJDB19081). 

MLST analysis revealed that the JH62PP1 and JH62PN strains isolated from the patient’s pus and nasal swab specimens at the initial visit, as well as those (JH62F, JH62M, and JH62B1) from nasal swab specimens of the father, mother, and older brother, were identified as MSSA sequence type (ST) 2233 (Appendix Figure 1). ST2233 belongs to clonal complex (CC) 188, as determined by eBURST analysis of PHYLoViZ version 2.0 web tool (https://www.phyloviz.net). JH62PP2, isolated from the patient’s pus at the follow-up visit, belonged to ST1, and JH62B2, isolated from the younger brother’s nasal swab, ST188; their STs were distinct from ST2233, which was the ST of the suspected strain of household transmission. In the case of *S. aureus*, the single-nucleotide polymorphism cutoff value for excluding patient-to-patient transmission in an outbreak setting is considered to be <15 within the previous 6 months (*7*). Therefore, all ST2233 isolates from the 4 family members were found to be very closely related, confirming that the causative clone was transmitted within the family.

We also investigated the virulence, antimicrobial-resistance, and disinfectant-resistance genes by whole-genome sequencing analysis (Table). Five ST2233 isolates possessed multiple virulence genes: Panton-Valentine leukocidin genes *lukS-PV* and *lukF-PV*; staphylococcal enterotoxin genes *seg*, *seh*, *sei*, *sem*, *sen*, *seo*, *ses*, and *seu*; exfoliative toxin gene *ete*; and some antimicrobial-resistance genes, including β-lactam resistance gene *blaZ* and quinolone resistance–determining region mutations GrlA S80Y and GyrA S84L. However, we did not detect the mupirocin-resistance gene *mup* or any *qac* homologs, such as *qacA*, *qacB*, *qacE*, *qacG*, and *qacH*, which are known as disinfectant-resistance genes and known to reduce efficacy (*8**,**9*). We also investigated biofilm formation, which is associated with bacterial colonization and drug resistance. However, the biofilm-forming ability of these ST2233 strains was only slightly higher than that of the negative control FK300 (p<0.05) and not particularly strong (Appendix Figure 2).

**Table:** Genetic characteristics of strains isolated in study of skin infections caused by Panton-Valentine leukocidin and methicillin-susceptible *Staphylococcus aureus* in child, Japan*

ID	Source	ST	Relevant molecular characteristics
JH62M	Mother	ST2233	TGs: *pvl, ete, seg, seh, sei, sem, sen, seo, ses, seu*
			ARGs: *blaZ,* GrlA/S80Y, GyrA/S84L
			DRGs: none
JH62PP1	Patient	ST2233	TGs: *pvl, ete, seg, seh, sei, sem, sen, seo, ses, seu*
			ARGs: *blaZ,* GrlA/S80Y, GyrA/S84L
			DRGs: none
JH62PN	Patient	ST2233	TGs: *pvl, ete, seg, seh, sei, sem, sen, seo, ses, seu*
			ARGs: *blaZ,* GrlA/S80Y, GyrA/S84L
			DRGs: none
JH62B1	Older brother	ST2233	TGs: *pvl, ete, seg, seh, sei, sem, sen, seo, ses, seu*
			ARGs: *blaZ,* GrlA/S80Y, GyrA/S84L
			DRGs: none
JH62F	Father	ST2233	TGs: *pvl, ete, seg, she, sei, sem, sen, seo, ses, seu*
			ARGs: *blaZ,* GrlA/S80Y, GyrA/S84L
			DRGs: none
JH62B2	Younger brother	ST188	TGs: none
			ARGs: none
			DRGs: none
JH62PP2	Patient (second time)	ST1	TGs: *sea, seh, sek, seq, ses*
			ARGs: *blaZ, ant(**9**)-Ia, erm*(A)
			DRGs: none

*ARG, antimicrobial-resistance gene; DRG; disinfectant-resistance gene; ID, identification; ST, sequence type; TG, toxin gene.

ETE is a recently identified toxin that specifically degrades desmoglein-1 in the epidermis of many mammal species, including humans (*10*). A PVL and ETE double-positive strain is very rare; only 1 case has been reported, from necrotizing fasciitis (*11*). We have not found other reports of PVL and ETE double-positive *S. aureus* belonging to CC188, the most frequently isolated clone from healthy adult skin in Japan.

## Conclusions

SSTIs caused by *S. aureus* are known to recur. A large multicenter cohort study of 959 staphylococcal SSTI cases revealed that 16.4%–19.0% of patients experienced >1 recurrence within a 12-month follow-up period (*1*). Intrafamilial transmission of *S. aureus* in patients with SSTIs is also known; Rodriguez et al. reported that among 163 pediatric patients with community-associated *S. aureus* SSTIs, intrafamilial strain relationships were observed in 105 (64%) families (*12*). Decolonization is occasionally attempted for all family members of patients with relapsing staphylococcal SSTIs; well-known decolonization techniques are nasal decolonization by mupirocin ointment and topical body decolonization by chlorhexidine (*3*) and sometimes bleach baths, in which the patient bathes in a bathtub filled with diluted hypochlorous acid water (*13*). Although we applied all 3 techniques to our patient and her family members, they were ineffective. Detailed strain analysis showed that the strains did not harbor resistance genes to mupirocin or disinfectants, and resistance to them was not the cause of their insufficient efficacy. Further strategies to control relapsing staphylococcal SSTI are required.

In summary, we encountered a child with relapsing SSTI caused by a PVL and ETE double-positive MSSA ST2233 strain that was refractory to decolonization procedures. Further investigation will reveal the clinical significance of the ST2233 strain and effective techniques for decolonization. Clinicians should be aware of the possibility of disinfectant resistance in SSTIs caused by MSSA infections.

Appendix Additional information about skin infections caused by Panton-Valentine leukocidin and methicillin-susceptible *Staphylococcus aureus* in child, Japan.
