# Supplementary material for: Skin Infections Caused by Panton-Valentine Leukocidin and Methicillin-Susceptible Staphylococcus aureus in Child, Japan
Source: Emerg Infect Dis. 2025 Jun;31(6):1227–30. doi: 10.3201/eid3106.241955 (PMC12123911; doi:10.3201/eid3106.241955)
Supplement: Appendix — Additional information about skin infections caused by Panton-Valentine leukocidin and methicillin-susceptible Staphylococcus aureus in child, Japan. [file 24-1955-Techapp-s1.pdf]

Article DOI: https://doi.org/10.3201/eid3106.241955

EID cannot ensure accessibility for supplementary materials supplied by authors. Readers who have difficulty accessing supplementary content should contact the authors for assistance.

Skin Infections caused by Panton-Valentine Leukocidin and Methicillin-susceptible *Staphylococcus aureus* in Child

Appendix

Appendix Table. Antimicrobial susceptibility of *Staphylococcus aureus* (JH62PP1)

Antibiotics	MIC	Susceptibility
Clindamycin	≤0.5	S
Levofloxacin	≥8	R
Gentamicin	≤2	S
Trimethoprim/sulfamethoxazole	≤19	S
Vancomycin	= 1	S
Linezolid	= 1	S

MIC, minimum inhibitory concentration

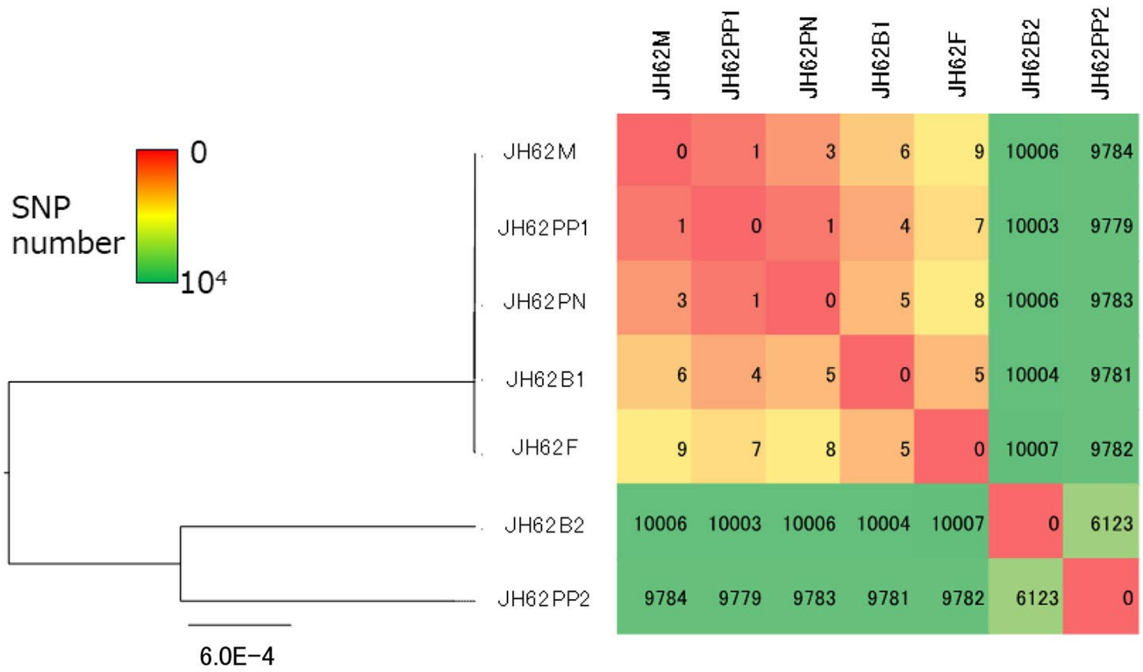

Appendix Figure 1. Phylogenetic relationships of *S. aureus* strains from a child experiencing recurrent skin infections and her family members. We constructed a core-genome–based maximum-likelihood recombinant-free phylogenetic tree of 7 *S. aureus* strains using RAxML-NG version 0.9.0 (https://github.com/amkozlov/raxml-ng) at the genetic testing registry (GTR)+G4 model and 100 bootstrap replicates. We calculated pairwise single-nucleotide polymorphism distances in the final aligned sequences using *snp-dists* version 0.8.2 (https://github.com/tseemann/snp-dists). We detected virulence factor genes and antimicrobial resistance genes using ABRicate version 1.0.1 (https://github.com/tseemann/abricate). The scale bar represents the number of nucleotide substitutions per site.

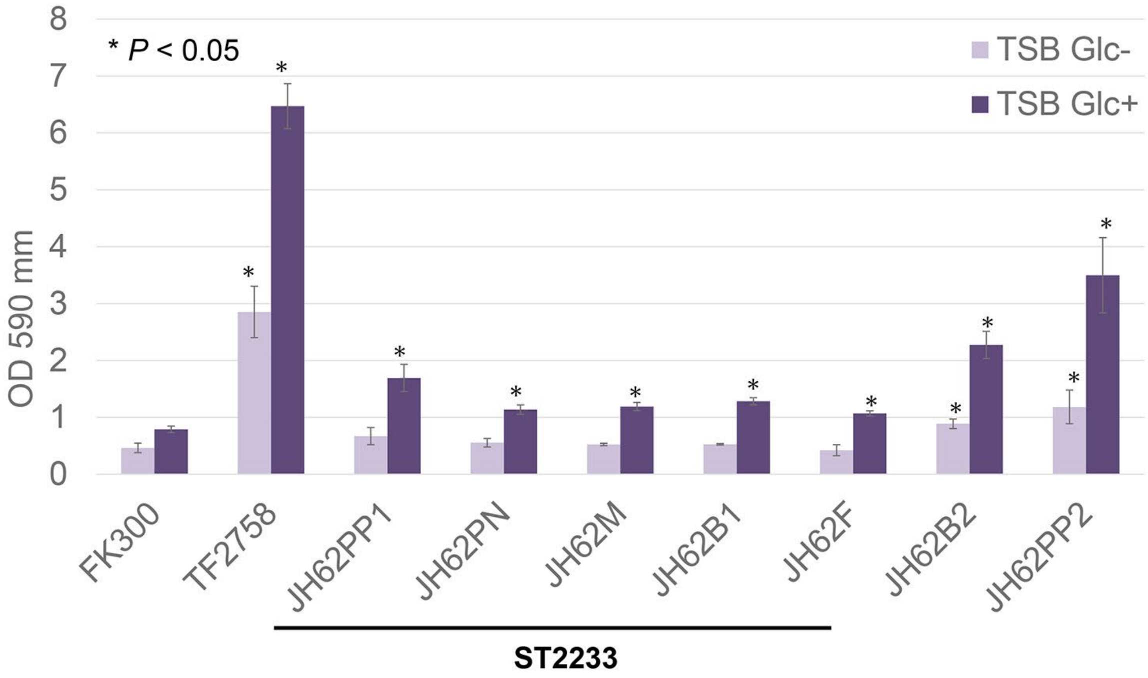

Appendix Figure 2. Biofilm-formation assay of *S. aureus* strains from the patient and family members. Biofilm assay was performed as previously described (Kutsuno S. et al., *Front Microbiol*. 2022 13:1101545). FK300 strain was used as a negative control, and the TF2758 strain was used as a positive control in this assay. Bars indicate mean values and error bars indicate standard error of the mean (n = 3).
